# Psychopathology in adult transgender people

**DOI:** 10.1192/j.eurpsy.2021.151

**Published:** 2021-08-13

**Authors:** G. Castellini, J. Ristori, T. Steensma

**Affiliations:** 1 Department Of Health Science, University of Florence, Florence, Italy; 2 Andrology, Women’s Endocrinology And Gender Incongruence Unit, University of Florence, Florence, Italy; 3 Center Of Expertise On Gender Dysphoria, Amsterdam UMC, VU University Medical Center, Amsterdam, Netherlands

## Abstract

Transgender people represent a broad spectrum of individuals that transiently or persistently identify with a gender different from the one assigned at birth (APA, 2013). Transgender healthcare issues have become an increased matter of interest over the last years, as shown by the growth of publications on the subject and by the increase of referrals reported worldwide in all age groups. However, transgender people report encountering numerous obstacles in accessing and receiving appropriate health care with professionals being described as not properly trained on specific gender issues. Considering the diversity and complexity of the gender spectrum in the different age groups and in light of the recent changes in the formal psychiatric classification, mental health providers play a critical role in meeting the needs of gender non-conforming children, adolescents and adults, according to individualized paths. This course has the following aims: (1) learn the principles of assessment of gender incongruence in childhood, adolescence and adulthood; (2) address co-occurring psychopathology (if present); (3) identify different treatment paths according to age and individualized psychological and/or medical needs; (4) work in a multidisciplinary team in line with an integrated model.

**Disclosure:**

No significant relationships.

